# Three-dimensional hard and soft tissue imaging of the human cochlea by scanning laser optical tomography (SLOT)

**DOI:** 10.1371/journal.pone.0184069

**Published:** 2017-09-05

**Authors:** Nadine Tinne, Georgios C. Antonopoulos, Saleh Mohebbi, José Andrade, Lena Nolte, Heiko Meyer, Alexander Heisterkamp, Omid Majdani, Tammo Ripken

**Affiliations:** 1 Biomedical Optics Department, Laser Zentrum Hannover e.V., Hannover, Germany; 2 Cluster of Excellence “Hearing4all”, Hannover, Germany; 3 Department of Otorhinolaryngology, Head and Neck Surgery, Hannover Medical School, Hannover, Germany; 4 Biofabrication for NIFE, Hannover, Germany; 5 Institute of Quantum Optics, Leibniz University of Hanover, Hannover, Germany; Universitat Zurich, SWITZERLAND

## Abstract

The present study focuses on the application of scanning laser optical tomography (SLOT) for visualization of anatomical structures inside the human cochlea *ex vivo*. SLOT is a laser-based highly efficient microscopy technique which allows for tomographic imaging of the internal structure of transparent specimens. Thus, in the field of otology this technique is best convenient for an *ex vivo* study of the inner ear anatomy. For this purpose, the preparation before imaging comprises decalcification, dehydration as well as optical clearing of the cochlea samples *in toto*. Here, we demonstrate results of SLOT imaging visualizing hard and soft tissue structures with an optical resolution of down to 15 μm using extinction and autofluorescence as contrast mechanisms. Furthermore, the internal structure can be analyzed nondestructively and quantitatively in detail by sectioning of the three-dimensional datasets. The method of X-ray Micro Computed Tomography (μCT) has been previously applied to explanted cochlea and is solely based on absorption contrast. An advantage of SLOT is that it uses visible light for image formation and thus provides a variety of contrast mechanisms known from other light microscopy techniques, such as fluorescence or scattering. We show that SLOT data is consistent with μCT anatomical data and provides additional information by using fluorescence. We demonstrate that SLOT is applicable for cochlea with metallic cochlear implants (CI) that would lead to significant artifacts in μCT imaging. In conclusion, the present study demonstrates the capability of SLOT for resolution visualization of cleared human cochleae *ex vivo* using multiple contrast mechanisms and lays the foundation for a broad variety of additional studies.

## Introduction

Imaging the functional anatomy of the human inner ear is of high interest for the study and therapy of hearing disorders [1–3]. Here, imaging modalities that enable non-destructive visualization of the three-dimensional structure of the cochlea are of special interest. There are two fundamental modalities of imaging the human cochlea: *in vivo* and *ex vivo*. *In vivo* measurements are performed on patients before or after cochlear implant (CI) insertion. Volumetric data is then used to inform the insertion procedure and to assess implant placement after insertion. It has been shown that nuclear magnetic resonance (NMR) tomography as well as X-ray computed tomography (CT) are particularly suited for this task [4–6].

*In vivo* studies have inherent limitations concerning the obtainable resolution since the cochlea has to be imaged inside the human skull, which is much larger than the volume of interest. In contrast, *ex vivo* studies allow explantation and preparation of the cochlea sample. This enables higher resolution imaging, since undesirable bony surroundings can be removed before imaging. Thus, high resolution micro computed tomography (μCT) imaging of explanted samples is possible [7,8]. However, μCT is mostly limited to X-ray absorption contrast, which is an excellent tool for studying the volumetric shape of the cochlea. More information about the soft tissue can be collected by phase contrast tomography [9], which is, however, not conducive to fluorescence labelling, a technique that is widely used in the biomedical sciences. Moreover, it suffers from severe image artifacts when metallic cochlear implants are located within the sample [8].

Volumetric imaging techniques that use visible light for illumination are able to overcome some of these limitations. However, in contrast to X-ray based imaging techniques, they need transparent samples that can be obtained from explanted cochleae by using well-known and established decalcification and clearing protocols [1,10,11]. In particular, the use of fluorescence as a contrast mechanism provides insight into the functional anatomy of the cochlea. For example, laser scanning confocal microscopy was used to visualize the inner hair cells and spiral ganglion cells in an optically cleared murine cochlea [1,12]. However the field of view in all three spatial dimensions is limited by the objective. This can be overcome by scanning thin-sheet laser imaging microscopy (sTSLIM) while maintaining high optical resolution [13–16].

In recent years, Scanning Laser Optical Tomography (SLOT) has emerged as a promising tool for volumetric imaging of mesoscopic samples with visible light [17–19]. SLOT provides enhanced collection efficiency in opposite to other optical imaging techniques as shown by Lorbeer *et al*. [17]. Furthermore, it does not suffer from shading artifacts, due to a full rotation of the sample during measurement. This way it is even possible to visualize the surface of non-transparent samples in 3D [20]. In this paper, we aim to show that SLOT enables volumetric imaging of cochlea samples, providing structural information consistent with μCT as well as information content. Furthermore, we generated volumetric images of an explanted cochlea containing a CI and show successful reconstruction. Although CT can also be applied *in vivo*, it must be noted that this work is only concerned with the *ex vivo* case since explanted cochleae are used throughout. The rest of the paper is organized as follows: first we will present the sample preparation for μCT and SLOT imaging, then we will describe the measurement process and compare the results of SLOT and μCT. After that, we present the volumetric datasets of the implanted sample, and finally, we discuss the results and give an outlook for potential applications and future work.

## Materials and methods

**Fig 1:**
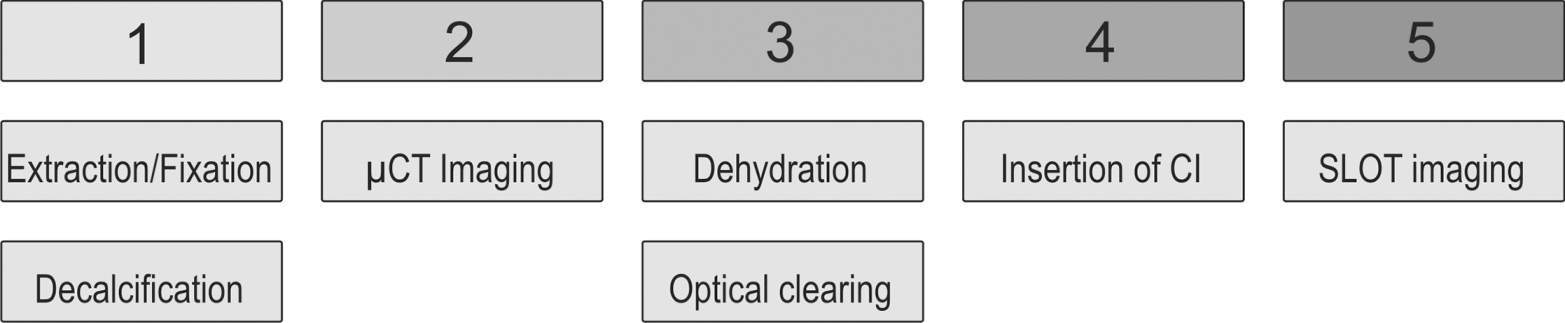
Schematic overview of the preparation protocol for human cochlear samples. 1) Extraction and fixation and decalcification, 2) μCT imaging, 3) dehydration and optical clearing 4) optional insertion of cochlear implant (CI), and 5) SLOT imaging.

During sample preparation the human cochleae went through different steps of a protocol as depicted in Fig 1. After explantation the cochleae were chemically fixed using 4% formol. Afterwards, optical coherence tomography (OCT) guided decalcification in combination with mechanical reduction of excessive bony tissue takes place which is described in detail elsewhere [10]. At this state, the μCT imaging was performed. For SLOT imaging, the sample has to be optically cleared. Therefore, dehydration as well as a matching of the refractive index is implemented. In a final step, the SLOT imaging was performed. All these sample preparation procedures are described in detail in this section. The study was performed in accordance with the Declaration of Helsinki, Good Clinical Practice, and applicable regulatory requirements. The use of human tissue in the form of four inner ears was approved by the ethics committee of the Hannover Medical School (No. 352–2008).

### Explantation and decalcification of human cochlea samples

For the presented study four fresh frozen explanted human temporal bone specimens from body donors at Hannover Medical School were used. These body donors gave their written consent during their lifetime providing their corpse for research purposes after their exitus. The explantation of the sample was performed within 24 hours after exitus of the donor. Mastoidectomy and middle ear opening were performed. The suprastructure of the stapes was removed, but the footplate was left intact. Temporal bones were then trimmed in cubic shape (1.5–2.0 cm length of each edge) with similar weight of 10–15 g. Furthermore, excess tissue was removed to simplify the procedure. The specimens were cut as follows: Lateral to tympanic membrane, medial to internal auditory canal, and inferiorly at the level of styloid process. Directly after explantation the fresh cochlea specimens were fixated in formaldehyde.

In order to enable fixation of inner ear structures after blue-lining the superior semicircular canal, a small hole of 1–2 mm diameter was created. Specimens were preserved in 4% formol at 4°C overnight. On the next day the specimens were restored in 50 ml of a 20% solution of ethylenediaminetetraacetic acid (EDTA). Following this, the samples were taken out and the softened part of bone was removed by 15 to 20 minutes of milling using the surgical system Aesculap microtron EC GD-631 (cutting burrs with sizes 2.8 and 1.8 mm, diamond burrs with sizes 1.8 and 1.0 mm). Here, OCT was used to analyze the thickness of the residual boney wall of the labyrinth organ (otic capsule) each time before further milling. The milling of all sides of the labyrinthine organ was processed until the thickness of the wall was homogeneously reduced to 300 μm [10].

During the process, the fragile specimens were held manually and care was taken not to damage the membranous labyrinth, keeping in place a thin bone shell. Afterwards, the samples were washed with phosphate buffered saline (PBS) three times and restored into fresh 20% EDTA medium. This procedure of milling was performed daily until specimen achieved circa 400 mg. Hence, decalcification typically took 10 days of preparation. The sample was then imaged with μCT and SLOT as described below.

### μCT imaging

Microscopic X-ray computed tomography (μCT) imaging was performed before SLOT imaging using a commercially available μCT system (phoenix nanotom® s, GE Measurement & Control, Germany). At this stage, cochlea samples had been decalcified and stored in PBS but not optically cleared. Before μCT imaging the sample was drained of fluid using a syringe. Before the measurement, in order to prevent desiccation, the sample was placed in a sealed polypropylene tube (Falcon Tube 15 ml, 62.554.502, Sarstedt AG & Co, Germany) that was filled partially with PBS to provide a humid atmosphere. Thereafter, the tube containing the sample was placed inside the μCT system and imaging was performed using a tube voltage of 80 kV and a current of 60 μA. Integration time for each projection was set to 2000 ms and a total of 2400 projections were acquired over a full revolution of the sample. After tomographic reconstruction, a volumetric dataset of the sample, consisting of 2284 x 2284 x 2304 voxels with an isotropic resolution of 4.5 μm per voxel was obtained.

### Optical clearing

**Fig 2:**
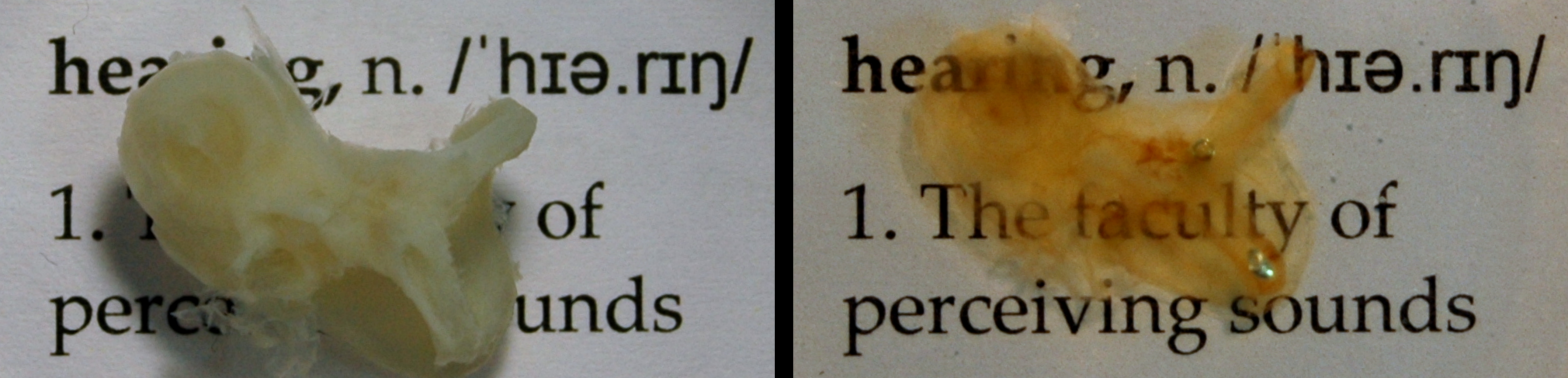
Illustration of optical clearing. Left: Photograph of a decalcified cochlea sample in PBS. The cochlea is not transparent to visible light. Right: The same cochlea after optical clearing and immersion in MSBB. The optical clearing process has made the sample transparent to visible light, thereby making the text behind the cochlea legible. Two air bubbles can also be seen in the semicircular ducts which are outside of the area of interest. Air bubbles could otherwise lead to artifacts in reconstructed SLOT images.

Before imaging with visible light, the sample must undergo an optical clearing procedure. We used a simple clearing protocol that is routinely employed for biological samples, whereby the sample is immersed in MSBB, a mixture of methyl salicylate (MS) and benzyl benzoate (BB). During the whole procedure of optical clearing, which is described in detail afterwards, the cochlea samples were located at a constant room temperature of about 20°C. First, the sample is dehydrated in an increasing ethanol series of 70%, 95% and three times 100%; here, every step lasted at least 24 h. For optical clearing, a MSBB mixture of refractive index n = 1.553 was produced. After dehydration the sample was first transferred into a mixture of 50% ethanol and the clearing liquid for 4 h. In following step this fluid was replaced with 100% MSBB clearing liquid with two more washing steps of 100% MSBB with 4 h each. After completion, the samples are transparent to visible light and ready for SLOT imaging (see Fig 2). It is worth noting that the optical clearing procedure is not difficult albeit time consuming. Multiple samples can be cleared in parallel, so that this should not be an issue for large sample sizes. Special measures must be taken if air bubbles occur in the cochlear ducts within the area of interest. These would lead to reconstruction artifacts in SLOT imaging and can be carefully removed by suction through a needle tip.

### Insertion of cochlear implant

**Fig 3:**
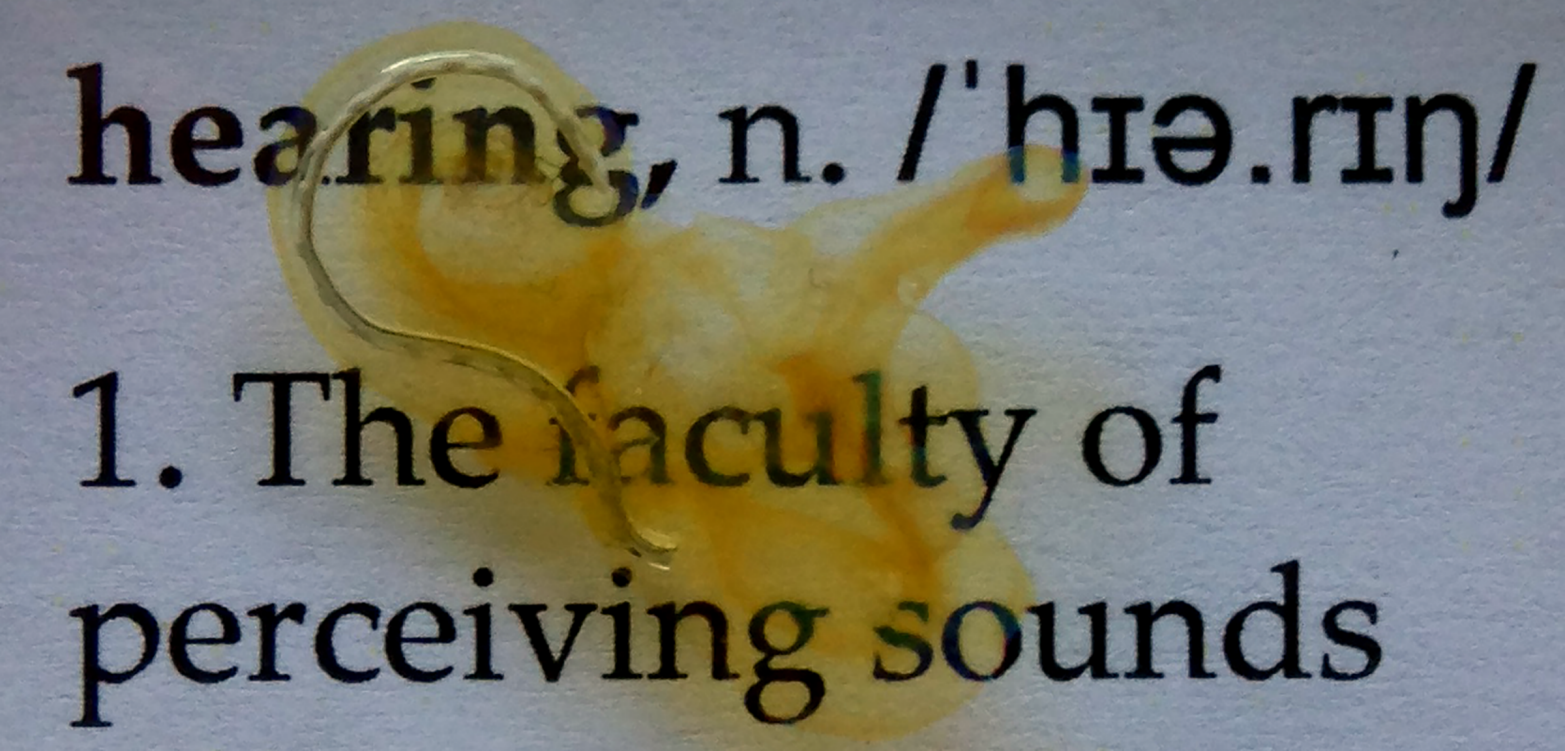
Optically cleared cochlea with cochlear implant Nucleus® CI 422 inserted. The cochlea sample shown here is different from the one depicted in Fig 2.

In order to assess the capability of SLOT for imaging of *ex vivo* cochlea after cochlear implant (CI) insertion, we prepared a cochlea sample with a CI inside. This sample was not the same that was imaged in the previous experiments. Implantation was performed by a medical expert on an optically cleared cochlea sample under a surgical microscope. For that, the round window was opened and the implant Nucleus® CI 422 (Cochlear™, Australia) was inserted. After insertion was deemed complete, the protruding part of the implant was cut leaving behind an optically cleared cochlea sample with the implant completely inside (see Fig 3). The sample was then imaged using the SLOT setup as described below.

### SLOT imaging

**Fig 4:**
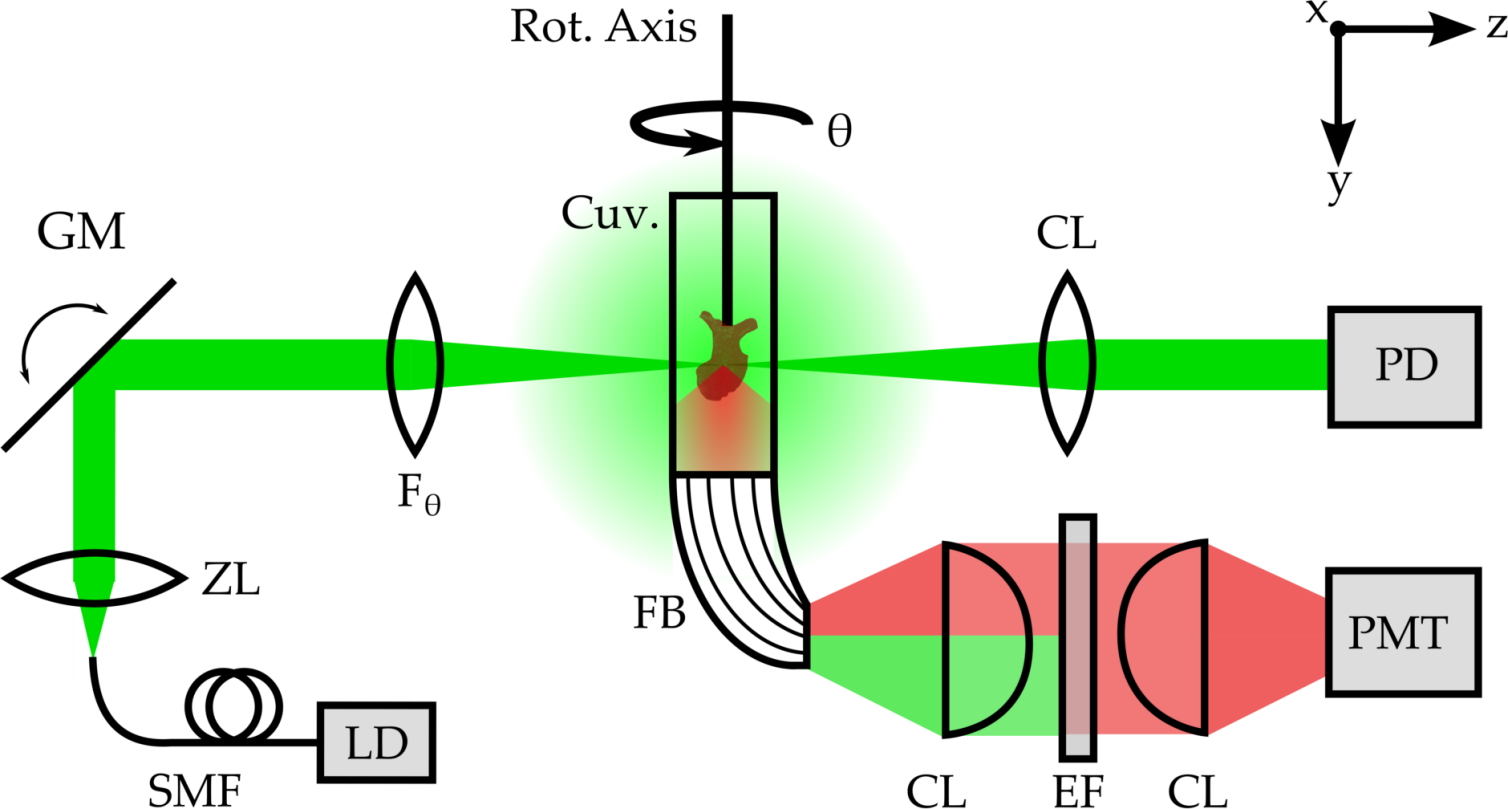
Schematic of the SLOT experimental setup. A laser diode (LD) is coupled into the setup using a single mode optical fiber (SMF). At the end of the fiber, the light is collimated and its beam diameter is adjusted using a motorized zoom lens (ZL). The beam is focused on the rotational axis of the system and scanned across the sample in x-y-direction using a combination of 2-axis galvanometric scanning mirror (GM) and a telecentric Fθ-lens. The sample itself is inside a cuvette filled with MSBB and attached to the axis of rotation. Transmitted light is detected with a photodiode (PD). Fluorescence light is collected with a fiber bundle and filtered with a fluorescence emission filter (EF). Thus, scattered light is blocked before the signal is measured with a photomultiplier Tube (PMT). For each scan a digital projection image is formed in a computer and the sample is rotated for a full revolution in small angular steps.

Since SLOT is a relatively novel technique without widespread use, we will provide a brief description of the setup; a detailed explanation can be found elsewhere [21]. In principle, SLOT is computed tomography with visible laser light as the source of illumination. Fig 4 shows a schematic of the experimental setup. A 520 nm laser diode (LD-520-50SG, Roithner Lasertechnik GmbH, Austria) is coupled into the setup using a single mode optical fiber. Light exiting the fiber tip is collimated and the beam diameter is adjusted using a 10x motorized zoom lens (H10Z1218MP, CBC (AMERICA) Corp., USA). With a combination of a two-axis galvanometric scanner (ProSeries II Scan Head, Cambridge Technology Inc., Germany) and a telecentric F_θ_-lens (S4LFT0080/121, Sill Optics GmbH & Co., Germany) the beam is focused to a needle beam. It is scanned across the sample, which is attached to a rotation stage inside a sample chamber. The sample chamber consists of a glass cuvette (700.016-OG, Hellma GmbH & Co., Deutschland) with planar entry windows and is filled with the clearing solution MSBB. Transmitted light is captured by a photo diode (PDA100A, Thorlabs GmbH, Germany). Thus, this detection channel provides information about light extinction due to absorption and residual scattering. At the bottom of the glass cuvette, using an optical fiber bundle, scattered light and excited autofluorescence is collected and filtered using a 570 nm long pass fluorescence filter (46–061 OG-570 1 Inch Long Pass Filter, Edmund Optics, Germany). This signal is detected with a photomultiplier tube (PMT, R3896, Hamamatsu Photonics K.K., Japan). Hence, this detection channel measures the autofluorescence of the sample when excited with 520 nm. The photodiode and PMT channels are acquired simultaneously.

The specimen is attached to a mechanical rotation stage inside the sample chamber and rotated in equidistant angular steps for a full revolution. For each rotational position a projection image is acquired by scanning the sample in the x-y-plane. After all projection images are captured, volumetric reconstruction of the sample is achieved using the common filtered back projection algorithm for parallel beam CT. For this, a set of custom ImageJ macros is used in combination with the IMOD package [22]. With this, a volumetric dataset for each detection channel is reconstructed.

Projection images were captured with a pixel resolution of 2000 x 1499 pixels and 2400 rotational increments. Thus the volumetric reconstruction resulted in a dataset of 2000 x 2000 x 1499 voxels with a spatial dimension of 5.3 μm^3^ for each voxel. Before the measurement, the numerical aperture of the scanning needle beam had to be adjusted to NA ≈ 0.016 so that the whole sample was within the depth of field of the imaging system. This resulted in a diffraction limited optical resolution of approximately 15 μm^3^ per voxel. Spatial overlap of the voxels was chosen in compliance with the Nyquist criterion.

### Image registration

Due to practical limitations, the sample orientation and voxel scaling could not be chosen identically for SLOT and μCT imaging. Thus, the resulting datasets had to be aligned before a comparison was possible. The alignment process was carried out in two steps. First, a coarse alignment was performed manually in ImageJ. By using global cropping, scaling and rotating operations the SLOT dataset was aligned to the μCT dataset so that it showed roughly the same size and orientation of the cochlea sample. In the second step, the fine alignment was carried out automatically using the open source software 3D Slicer [23] allowing only translation, rotation and scaling operations. No other distortions (e.g. skew) were allowed for the image registration process. Hence, the automatic registration matches the spatial alignment and the scaling of the datasets, but leaves any other differences unaltered. After this procedure, the SLOT and μCT datasets were aligned and could be compared with respect to the structures of the cochleae.

## Results and discussion

### Sample preparation

It has been reported by Brunschwig et al. that shrinkage of about 15% of the cochlear soft tissue can occur during fixation process [24]. We didn’t analyze this behavior in detail but we could not observe significant changes in volume of the decalcified tissue during this first preparation step. The clearing procedure, or more specific the dehydration step, can cause shrinking of up to 50% in volume. The application of the clearing agent causes a subsequent swelling of the sample so that in the end, the total shrinkage is roughly about 10% depending on the dehydration liquid and the clearing agent. However, this seems to be limited to soft tissues as described by Becker et al. [25]. The sample as depicted in Fig 2 has been measured: Both images are equal in pixel size. For the measurements specific landmarks on both images have been marked and analyzed using ImageJ. It appeared that no significant change in size could be observed. This might be explained due to the fact that the samples used here mainly consist of hard tissue which counteracts the shrinking. Hence, there is good comparability of the native tissue samples before its preparation and the structured detected with SLOT imaging regarding the anatomical dimensions.

### SLOT imaging of human cochlea

**Fig 5:**
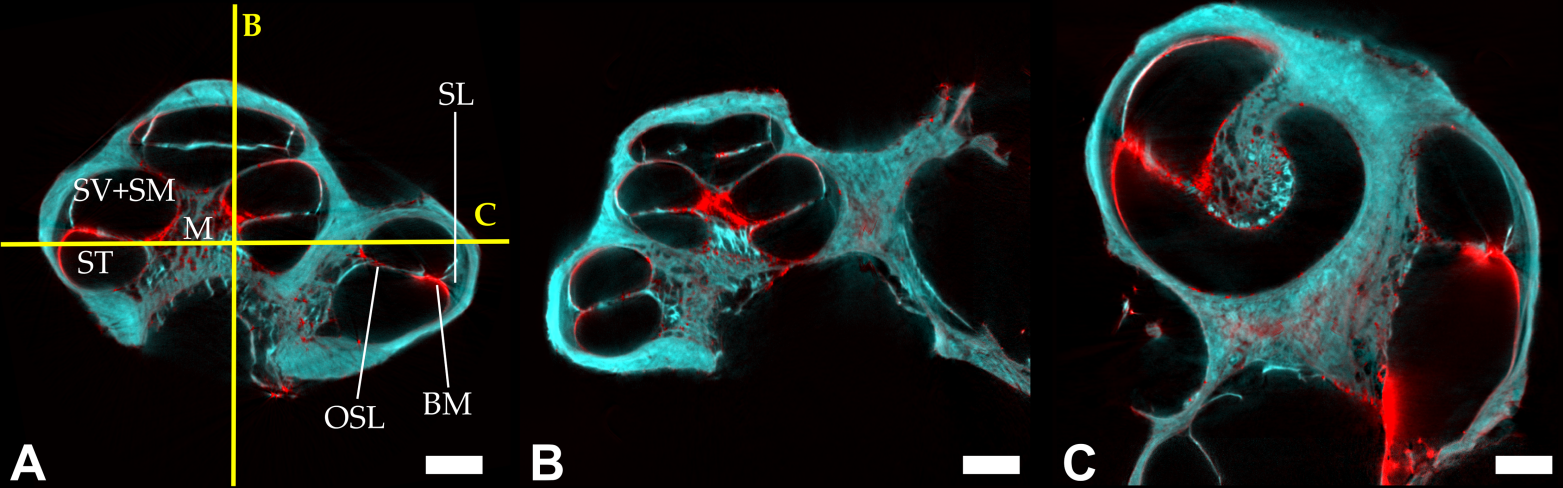
SLOT cross section images of the human cochlea. Figures A to C show three orthogonal planes of the 3D dataset. The detection channels have been superposed: Autofluorescence (cyan) and extinction (red). The following anatomical structures can be clearly identified: scala tympani (ST), scala vestibuli and scala media (SV+SM), modiolus (M), basilar membrane (BM), the spiral ligament (SL), and osseous spiral lamina (OSL). Here, scala vestibuli (SV) and scala media (SM) are labeled as a unity, because Reissner’s membrane is not visible to distinguish between the two ducts and clearly localize them. The scale bar corresponds to 1 mm.

Imaging of the human cochlea with Scanning Laser Optical Tomography (SLOT) gives a detailed insight to the anatomic structure of the inner ear (see Fig 5). In Fig 5A, a cross section of the volumetric data set is shown. Here, the two detection channels are depicted as an overlap image: While the autofluorescence has been colored cyan, the extinction can be seen in red. In turn, Fig 5B and 5C show the orthogonal planes which are outlined by yellow lines in Fig 5A.

The SLOT images show that the cochlear anatomy can be visualized in detail. Various anatomical structures can be observed within the 3D dataset: the scala tympani (ST), scala vestibuli (SV) and scala media (SM), modiolus (M), as well as basilar membrane (BM) are clearly visible. Furthermore, the spiral ligament (SL) and osseous spiral lamina (OSL) can be located. Certainly, the structure of Reissner’s membrane, anatomically located between SC and SM, cannot be located within the human cochlea sample (see Fig 5). As an explanation, two different approaches have to be taken into account: the technology of SLOT imaging and the procedure of sample preparation. In terms of the former aspect, the SLOT images clearly show that the imaging resolution of SLOT is really high with of 4.5 μm per voxel. Hence, even membranes can be detected as single structures in detail. A loss of resolution would make for an even thicker appearance of fine structures. Furthermore, sensitivity might be a technical factor to cause this circumstance. However, even fine structures like Reissner’s membrane could be clearly identified within the datasets during studies of SLOT imaging of guinea pig and murine cochlea samples. As a conclusion, due to the fact that technical reasons can be excluded as cause the preparation protocol for the human cochlea samples should be analyzed in detail. Here, an optimization of cochlea handling could submit to visualize the whole intra-cochlear tissue structures in future studies.

The analysis of the signal generated by the two detection channels of SLOT reveals that the autofluorescence signal appears dominantly and unspecifically. It can be seen all over the sample and allows for the visualization of tissue structures, e.g. the spongy appearance of the modiolus. In contrast, the extinction dominates at the boundary of the tissue. This is most likely due to refractive effects caused by a slight refractive index mismatch between the sample and the surrounding medium.

### Comparison of SLOT images with μCT

**Fig 6:**
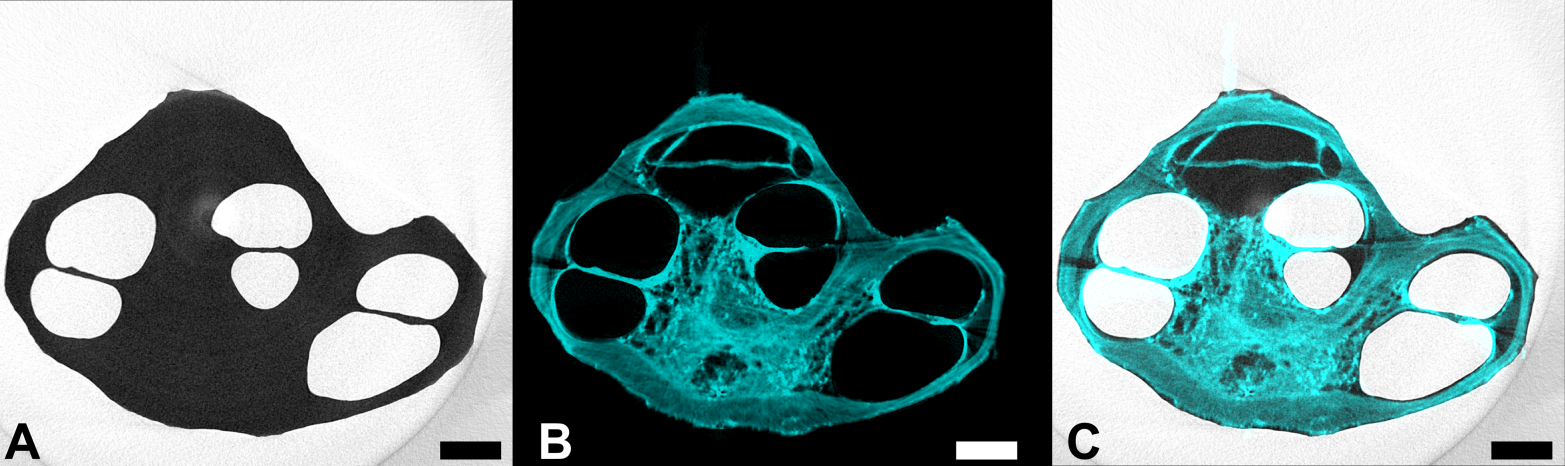
μCT and SLOT images of the identical human cochlea sample. The two data sets have been aligned after imaging: A μCT image in grey scale, B SLOT autofluorescence image in cyan, and C superposition of the two data sets for detailed comparison. It can be seen that one cochlear duct is not visible in the CT images which is most likely due to residual water inside the duct as a consequence of incorrect sample preparation. The scale bar corresponds to 1 mm.

The comparison of the SLOT with μCT images of the identical human cochlea sample is depicted in Fig 6. For this purpose, the data sets have been digitally aligned after imaging as previously described. Fig 6A shows the inverted μCT image. This means, that the darker the grey value the higher the absorption of the sample. It is observable, that there is a high contrast between the bony cochlea structure and the ducts which have been air-filled during μCT imaging. However, the dynamic range is not sufficient for displaying the anatomical structures in detail. This might be corrected by carefully adjusting CT imaging parameters, i.e. cathode voltage and current. However, this was not the aim of this study and the images shown in Fig 6 are of similar quality as in literature regarding their anatomic information content [8].

While in Fig 6B the SLOT image is shown again, Fig 6C introduces a superposition of the two data sets. By comparison, it is clearly visible that the cochlear ducts close to the apex have not been detected by the μCT system. This is probably caused by residual PBS inside the duct due to incorrect sample preparation. A damage of the specimen can be excluded because the entire burrow system can be seen in the SLOT images, which were produced afterwards.

### SLOT imaging of human cochlea with inserted CI

**Fig 7:**
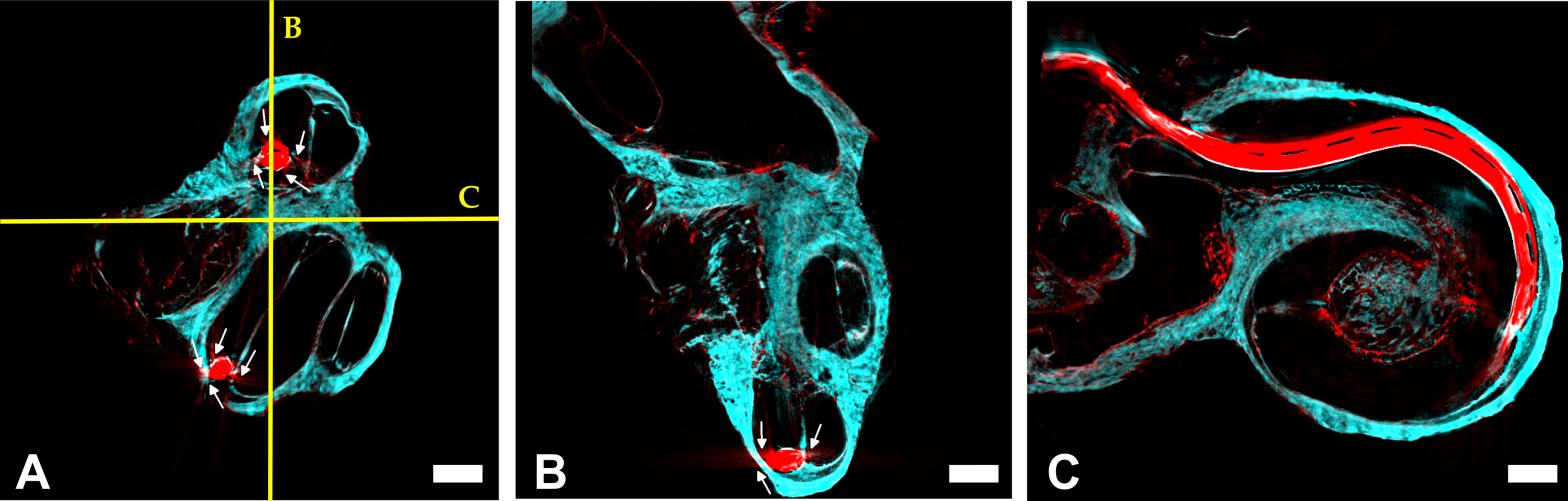
SLOT images of the human cochlea with cochlear implant electrode (Nucleus® CI 422) inserted. Figures A to C show three orthogonal planes of the 3D dataset. The detection channels have been superposed: Autofluorescence (cyan) and extinction (red). The white arrows indicate radial artifacts due to the reflections at the metal surface. The scale bar corresponds to 1 mm.

The results of SLOT imaging of the human cochlear after insertion of a cochlear implant (CI) are shown in Fig 7. Again, a cross section of the volumetric data set is shown in Fig 7A. The two detection channels are again depicted as an overlap image: The autofluorescence in cyan and the extinction depicted in red. Once again, Fig 7B and 7C show orthogonal planes which are outlined by the yellow lines in Fig 7A.

Due to the implant, radial artifacts occur during imaging of the inserted cochlea sample. They appear in form of concentric rays around the implant and can be seen in the extinction channel (red) (see white arrows in Fig 7). Compared to the X-ray tomography of a cochlea image in literature, the artifacts are relatively minor (see images in Postnov *et al*. [8]). While artifacts during μCT imaging arise mainly due to scattering at the metal surface, artifacts during SLOT imaging are caused by direct reflections that let the implant surface appear very bright only in few projections. These lead to so called streak artifacts. In their publication about μCT imaging of a human cochlea with inserted CI, Postnov *et al*. state that the image quality becomes so heavily decreased because of the CI that the authors had to merge the tomographic datasets with and without cochlear implant in order to reconstruct the precise position of the CI [8].

Hence, SLOT offers the great advantage of imaging a cochlear implant directly at its operating place, albeit *ex vivo*. Despite minor reconstruction artifacts, SLOT allows to analyze the measure of the implant after insertion in detail. Although SLOT is limited to explanted and optically cleared samples this opens up a variety of interesting studies: Under the assumption that there is no significant change in the mechanical properties of the sample due to its preparation, SLOT imaging enables the visualization of case studies like comparisons of different types of cochlear implants. Moreover it allows for volumetric measurements of the precise position, in form of space between implant and functional membranes (e.g. basilar membrane) or insertion depth. Therefore, further studies of the mechanical properties after sample preparation should be performed. The best position of the electrode would be near to modiolus (M). However, in our datasets the electrode is close to the lateral wall along most of its length. This analysis would allow to evaluate the insertion process of physicians in training. The fact that the sample is optically cleared can offer an extra advantage by controlling the insertion procedure via a surgical microscope. This way, physicians in training could practice the CI insertion and check, whether tissue damage occurred due to effects like e.g. tip fold over, bending or even tissue penetration. However it must be noted that the immersion in clearing liquid as well as the decalcification changes the mechanical properties of the cochlea such as friction and plasticity.

Finally, SLOT is a technique which provides the possibility to simultaneously measure different well-established contrast mechanisms of optical microscopy. Thus, there is the possibility to additionally implement further steps of sample preparation. For example, a selective antibody staining of different anatomical structures and cell types is possible which has already been proven in and been published for murine samples [26]. This will enable to measure the position of the CI electrodes with respect to the neurons which have to be electrically stimulated by the CI can be studied and optimized. This could prove to be a valuable tool in studying and optimizing the performance of cochlear implants.

## Conclusion

We presented scanning laser optical tomography (SLOT) as a novel three-dimensional *ex vivo* microscopy technique with great potential in the field of otology. It enables to visualize hard and soft tissue of the explanted human cochlea *ex vivo* by using extinction and autofluorescence as contrast mechanisms. The results achieved with this technique are comparable to μCT images of explanted cochlea concerning anatomical information content. Beyond that, SLOT enables the use of well-established contrast mechanisms from light microscopy, especially fluorescence staining [26]. By that, a wide array of further studies is made possible. For example, this will allow performing selective antibody staining in order to visualize single cell and tissue types. With that, a more detailed anatomical and functional analysis of the human cochlea *ex vivo* is conceivable. These possibilities are especially interesting since SLOT imaging is applicable in the presence of a metallic cochlear implant with reconstruction artifacts markedly decreased in comparison to X-ray computed tomography as reported by Postnov *et al*. [8].

The extent of imaging artifacts due to the metal parts of the implant when imaging a human cochlea with a CI inserted is decreased in comparison to μCT. This will allow for imaging of fluorescently labeled samples with inserted CI. Thereby the distance of electrodes to neurons, could be detected. In conclusion, the presented study has shown that SLOT is ideally suited for visualization of the internal structure of the (human) inner ear *ex vivo*.
